# Methodologies for Improving HDR Efficiency

**DOI:** 10.3389/fgene.2018.00691

**Published:** 2019-01-07

**Authors:** Mingjie Liu, Saad Rehman, Xidian Tang, Kui Gu, Qinlei Fan, Dekun Chen, Wentao Ma

**Affiliations:** ^1^College of Veterinary Medicine, Northwest A&F University, Xianyang, China; ^2^China Animal Health and Epidemiology Center, Qingdao, China

**Keywords:** CRISPR-Cas9, HDR, NHEJ, HDR enhancement, DSB, cell arrest, NHEJ inhibitors

## Abstract

Clustered regularly interspaced short palindromic repeats (CRISPR)-associated protein 9 (Cas9) is a precise genome manipulating technology that can be programmed to induce double-strand break (DSB) in the genome wherever needed. After nuclease cleavage, DSBs can be repaired by non-homologous end joining (NHEJ) or homology-directed repair (HDR) pathway. For producing targeted gene knock-in or other specific mutations, DSBs should be repaired by the HDR pathway. While NHEJ can cause various length insertions/deletion mutations (indels), which can lead the targeted gene to lose its function by shifting the open reading frame (ORF). Furthermore, HDR has low efficiency compared with the NHEJ pathway. In order to modify the gene precisely, numerous methods arose by inhibiting NHEJ or enhancing HDR, such as chemical modulation, synchronized expression, and overlapping homology arm. Here we focus on the efficiency and other considerations of these methodologies.

## Overview of CRISPR-Cas9 System

Clustered regularly interspaced short palindromic repeats (CRISPR) represents a family of DNA sequences in bacteria and archaea (Barrangou, 2015). This family is characterized by direct palindromic repeats, where sequences are the same in both directions, varying in size from 21 to 37 bp (Barrangou and Marraffini, 2014), interspaced by spacers, which have fragments gathered from viruses or phages that previously tried to infect the cell (Horvath and Barrangou, 2010; Morange, 2015). To appreciate this characteristic array, it is termed CRISPR (Jansen et al., 2002; Mojica and Rodriguez-Valera, 2016). CRISPR-associated (*cas*) genes are invariably located adjacent to a CRISPR locus (Jansen et al., 2002). The CRISPR-Cas system can be grouped into three types: type I, type II, and type III. In addition, there are 12 subtypes of the CRISPR-Cas system, which are based on their exclusive genetic content and structural differences (Makarova et al., 2015). *Cas1* and *cas2* are universal across types and subtypes, whereas *cas3, cas9, cas10* are signature genes for type I, type II, and type III, respectively (Makarova et al., 2011). Here in this review we only focus on type II. The CRISPR-Cas system functions as a defense system in bacteria and archaea against bacteriophage infection, conjugation, and natural transformation by degrading foreign nucleic acid that enters the cell (Marraffini, 2015). The CRISPR-Cas system involves three distinct mechanistic stages: adaptation, biogenesis, and interference (Marraffini and Sontheimer, 2010). The adaptation stage involves the integration of fragments of foreign DNA (termed “protospacers,” captured, excised, and inserted by Cas proteins) into the CRISPR array as new spacers. New spacers are usually added at the beginning of the CRISPR locus next to the leader sequence, creating a chronological record of viral infections (Sorek et al., 2013) and protecting the cell from further infection. During the biogenesis stage, the CRISPR array is transcribed as a single long transcript (termed “pre-crRNA”) containing much of the CRISPR array (Marraffini and Sontheimer, 2010) and is then processed and matured to produce CRISPR RNAs (crRNAs) with only one spacer sequence. As for the interference stage, the spacers in these crRNAs guide cas proteins to foreign DNAs and cleave them (van der Oost et al., 2009; Wiedenheft et al., 2012; Barrangou, 2013). The type II CRISPR-Cas system needs only *cas9* to execute immunity in the presence of an existing targeting spacer sequence (Sapranauskas et al., 2011). It requires two small RNAs: the crRNA and the trans-encoded crRNA(tracrRNA) (Deltcheva et al., 2011). TracrRNA forms a secondary structure that interacts with cas9 protein and it has a complementary region that enables itself to bind to pre-crRNA (Anders et al., 2014; Jinek et al., 2014; Nishimasu et al., 2014). The dsRNA formed between pre-crRNA and tracrRNA is then handled by RNase III to form mature crRNA guides that are used in genome editing. When crRNA and tracrRNA are combined together, they are collectively termed as guide RNA (gRNA) (Jinek et al., 2012). Another important short (3–5 bp) DNA termed protospacer adjacent motif (PAM) is required for targeting. PAM is a component of the invading virus or plasmid, but it is not a component of the bacterial CRISPR locus. Cas9 will not successfully bind to or cleave the target DNA sequence if it is not followed by the PAM sequence (Mojica et al., 2009). The first step in target recognition is the transient binding of Cas9 to PAM sequences within the target DNA, which promotes the unwinding of the two DNA strands immediately upstream of the PAM (Sternberg et al., 2014), the spacer sequence of the crRNA binds with the unwinded DNA (6–8 bp in length), then forms an RNA-DNA heteroduplex and triggers cleavage at the targeted site (Sternberg et al., 2014; Szczelkun et al., 2014). After recognition, the CRISPR-Cas9 system introduces a crRNA-specific DSB in the target sequence, which is further resolved either by homology-directed repair (HDR) or non-homologous end joining (NHEJ).

## NHEJ Pathway

**Figure 1:**
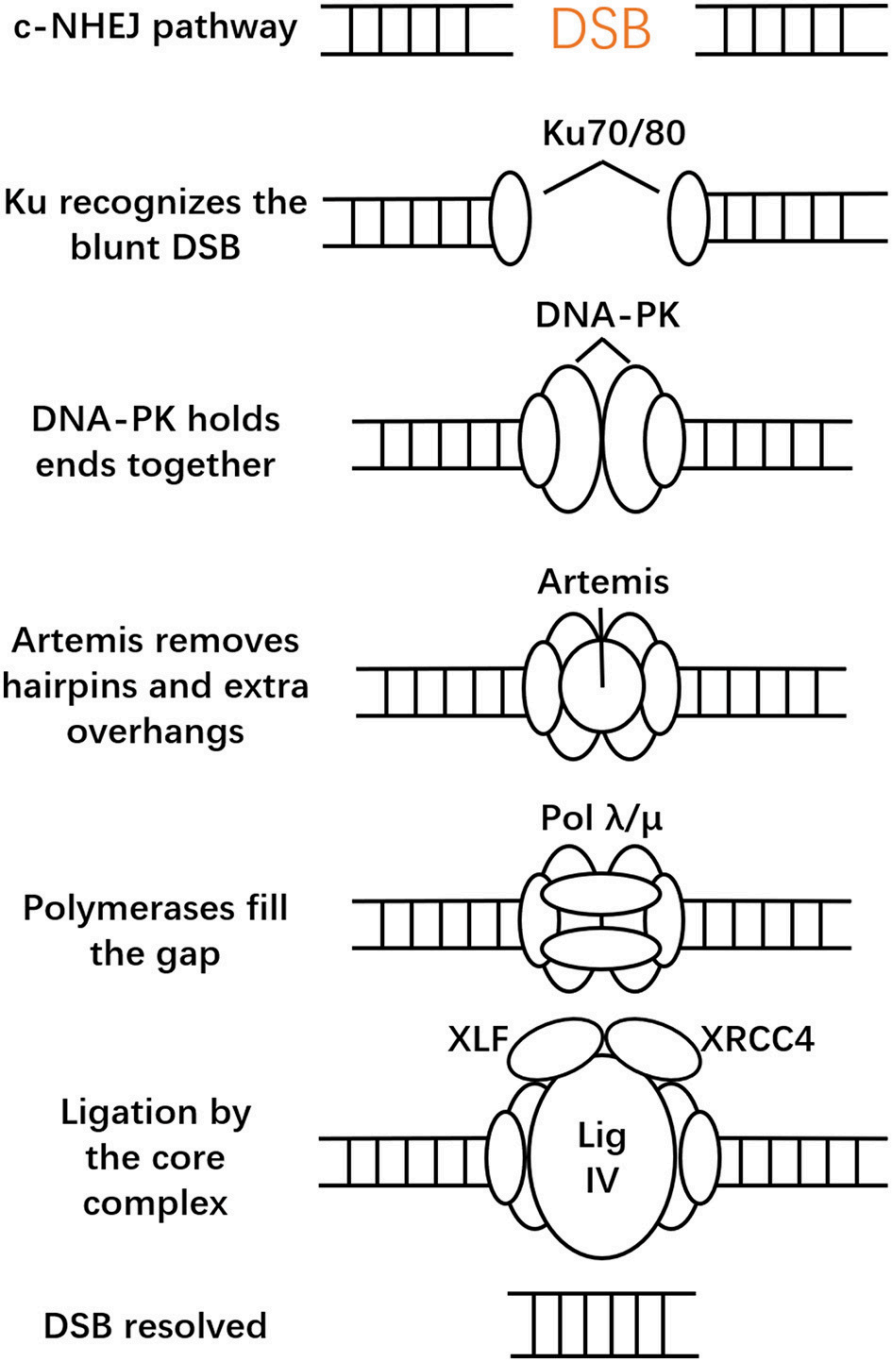
Canonical non-homologous end joining (c-NHEJ). After CRISPR-Cas9 introduced a DSB, NHEJ is initiated by the binding of the Ku heterodimeric complex. This then forms the core complex, which is considered to recognize broken ends and keeps them together. The ends will then be ligated by various ligases.

Typically, cells employ two main mechanisms to repair DSBs: classical NHEJ and HDR (Symington and Gautier, 2011). There are also many alternative error-prone DSB repair pathways: single-strand annealing (SSA) and breakage-induced replication (BIR) (Pardo et al., 2009; Jasin and Rothstein, 2013). SSA does not require a homologous template, and rejoining DNA ends with direct sequence repeats (Symington, 2014). BIR repairs one-ended DSBs, a process that is caused by the collapse of a replication fork (Mayle et al., 2015). When DSBs occur in cells, the first reaction is usually carried out in an NHEJ manner. Compared to other DNA repair and DNA recombination pathways, the NHEJ pathway is a robust, error-prone but predominant and fast pathway with high flexibility. It can recognize diverse end structures at DSBs and accomplish diverse repair results (Aravind and Koonin, 2001; Gu and Lieber, 2008; Salsman and Dellaire, 2017). NHEJ can be classified into two types: canonical NHEJ (c-NHEJ) and alternative NHEJ (alt-NHEJ), also called microhomology-mediated end-joining (MMEJ) (Bae et al., 2014). c-NHEJ is active throughout the cell cycle and stabilizes the DSB from translocations (Roth et al., 1995; Soutoglou et al., 2007). Based on different DNA ends, NHEJ is capable of employing different strategies. The whole process deals with assembling the core complex, which recognizes broken ends and keeps them together so that the following processing factors can act (Waters et al., 2014). The core complex is considered to include the Ku heterodimer (Ku80/70), the DNA-dependent protein kinase catalytic subunit (DNA-PKcs), DNA ligase IV, the X-ray repair cross-complementing protein 4 (XRCC4), the XRCC4-like factor (XLF, or Cernunnos). Ku is a heterodimer, composed of two subunits (70 and 83 kD), that recognizes and binds to blunt DSBs first (Walker et al., 2001). In c-NHEJ, Ku recruits DNA-PKcs to the DSB site and forms a very stable complex that remains bound to the end (Weterings et al., 2003). Their assembly activates the kinase activity of DNA-PK and orchestrates c-NHEJ (Davis et al., 2014; Radhakrishnan et al., 2014). DNA-PK phosphorylates a host of DNA damage response proteins and thus regulates c-NHEJ and DSB processing and recruits Artemis nuclease (Moshous et al., 2001). However, DNA-PK mostly phosphorylates itself, which is crucial in DSB processing (Neal et al., 2014). Artemis has 5′ to 3′ single-stranded DNA exonuclease activity and DNA-PKcs-dependent 5′ and 3′ endonuclease activity on hairpins and single-stranded overhangs (Moshous et al., 2001). Ku and DNA-PKcs alone can also promote multiple DNA end-processing activities at the break site. The X family of DNA polymerases (pol mu and pol lambda) adds missing nucleotides at the DSB ends (Daley et al., 2005; Paull, 2005). Next, the DSBs will be ligated by Ligase IV/XRCC4/XLF, which is regulated by DNA-PK. Ligase IV/XRCC4/XLF forms an extended filament that wraps and stabilizes DNA and stimulates ligation (Tsai et al., 2007; Andres et al., 2012). Recent research also showed that a newly identified PAXX (a paralog of XRCC4 and XLF), a member of the XRCC4 superfamily, is another important mediator of c-NHEJ, which interacts directly with Ku. In most cases DNA is repaired via the c-NHEJ pathway and its efficiency can approach nearly 90% (Yang et al., 2013; Dow et al., 2015), which constitutes the basis of CRISPR/Cas9 technology (Vartak and Raghavan, 2015). The NHEJ process is illustrated in Figure 1.

## HDR Pathway

HDR is a faithful repair pathway. It comes into action mainly in the S- or G2-phase of the cell cycle and requires homologous DNA sequences. Homologous recombination is the desired mechanism for precise genome editing, which only happens in the presence of a homologous duplex template to repair the broken site. When DSB occurs, pathway choice depends on end resection (Symington and Gautier, 2011). The MRE11-RAD50-NBS1 (MRN, MRX in yeast) complex recognizes dsDNA and first creates a nick 15–20 bp from the 5′-ends of the DSB (Symington, 2014). Exonucleases such as SGS1-DNA2 and EXO1 complete the resection step (Kim and Mirkin, 2018). It then moves into flanking dsDNA regions and recruits ataxia telangiectasia mutated (ATM) kinase, the key upstream kinase of DSB signaling (Falck et al., 2005), and interacts with CtIP (Makharashvili and Paull, 2015). MRN also tethers DNA ends, which increases its local concentration and thus facilitates ATM activation (Dupre et al., 2006).

The MRN complex consists of three subunits. MRE11 is a Mn^2+^ dependent nuclease involved in homologous recombination, telomere maintenance, and DNA DSB repair (Paull, 2015). SAE2 activates MRE11 for its dsDNA-specific endonuclease activity (Cannavo and Cejka, 2014) and regulates the resection step during appropriate stages of the cell cycle (Mathiasen and Lisby, 2014). RAD50 belongs to the structural maintenance of chromosomes (SMC) family, and contains ATPase activity (de Jager et al., 2001). RAD50 becomes dimerized and its DNA-binding activity is activated after ATP binding. Two MRE11 genes will then bind to the ATPase heads of the RAD50 homodimer, enabling itself to interact with RAD50 (Williams et al., 2008). RAD50 forms the core of MRN and uses its extended coiled-coil domain to tether DSB ends during HR (Williams et al., 2010; Hohl et al., 2011). NBS1 contains a Fork-Head associated (FHA) domain and BRCA1 C-terminal (BRCT) domain, binds MRE11 and recruits ATM, linking the core MRN activities to DNA damage response (DDR) proteins domains at its N terminus (Glover et al., 2004; Williams et al., 2009).

Histone variant H2AX is phosphorylated by ATM, which becomes γH2AX throughout the area surrounding the breakage within seconds after damage occurs (Rogakou et al., 1998). This sets off elaborate ubiquitylation and SUMOylation cascades to promote recruitment of BRCA1 (Morris et al., 2009) and 53BP1 (Stewart, 2009) but it is not crucial for the activation of ATM substrates such as CHK2 and p53 (Kang et al., 2005). Then MDC1, a large nuclear factor, directly binds to γH2AX and functions as a molecular scaffold that interacts with ATM and NBS1, promoting further MRN accumulation. In addition, MDC1 also helps ATM spread on DSB-flanking chromatin and furthers H2AX activation (Spycher et al., 2008). It also mediates the accumulation of many DDR factors, including 53BP1 and BRCA1 (Wang et al., 2002; Stucki and Jackson, 2006; Kim et al., 2007). ATM leads to phosphorylation of DDR cascades such as BRCA1, Chk2, p53, etc., (Shiloh, 2003; Lavin, 2008). DSBs also activate ataxia telangiectasia and RAD3-related protein (ATR). Full ATR activation requires not only itself and DNA damage sensors but also proteins that function as signal transducers and effectors, such as RPA, RAD17, TopBP1, Claspin, and Chk1. ssDNA overhangs will be coated by replication protein A (RPA) rapidly, and the ssDNA-RPA complex acts as a scaffold to attract ATR/ATR-interacting protein (ATRIP) (Zou and Elledge, 2003) and other DNA damage checkpoint kinases to trigger DDR (Chen and Wold, 2014). It will then be replaced with adenosine triphosphate (ATP)-dependent recombinase RAD51 (San Filippo et al., 2008) with the help of BRCA1 and BRCA2 as described above (Prakash et al., 2015). ssDNA can be generated by nuclease resection, such as the MRN-C-terminal binding protein interacting protein (CtIP) for short resection, and EXO1/BLM for long resection (Mladenov et al., 2016). In mammals, resection depends on CtIP and needs to be phosphorylated by CDK first (Huertas and Jackson, 2009). BRCA1 contributes to HR by colocalizing with MRN after DNA damage occurs and interacts directly with the resection factor CtIP (Sartori et al., 2007). BRCA1 assists RAD51 binding to ssDNA by evicting RPA (Zelensky et al., 2014) and promotes the recruitment of BRCA2 to DSBs through the bridging protein PALB2 (Sy et al., 2009). BRCA1 also appears to inhibit the resection suppressor 53BP1 (Bunting et al., 2010). RAD51 is a DNA strand-exchange protein that exists in mammalian cells and forms a filament referred to as the presynaptic complex (van der Heijden et al., 2007; Hilario et al., 2009). The assembly of a RAD51 nucleoprotein filament promotes homologous search by locating and pairing the 3′-overhang with a homologous duplex DNA and catalyzing strand invasion (termed single-end invasion, SEI) (Morrical, 2015; Ma et al., 2017). The two ends of the DSB are identical, but one end serves as the “first end,” which searches for the homologous sequence and forms a displacement loops (D-loops) structure while the other end waits for the latter process (Kim and Mirkin, 2018). Besides RAD51, DNA strand exchange also requires RAD54 and RDH54/TID1, which performs this step by stabilizing RAD51-ssDNA presynaptic filaments (Mazin et al., 2003).

Resolution of the exchanged DNA strands includes the Holliday Junction (HJ) pathway and the synthesis-dependent strand annealing (SDSA) pathway. Dissolution is the primary pathway for HJ resolution, which involves the BLM helicase-Topoisomerase IIIα-RMI1-RMI2 (BTR) complex. The BTR complex promotes branch migration of Holliday junctions (Karow et al., 2000) and also acts to suppress crossing over during homologous recombination (Wu and Hickson, 2003). Thus, this dissolution pathway gives rise exclusively to non-crossovers. The other pathways use structure-selective resolvases (SLX-MUS complex and GEN1) to process the exchange intermediates and can produce both crossover and non-crossover products (West et al., 2015). The SLX1 and MUS81-EME1 nucleases bind in close proximity on the SLX4 scaffold and process HJs (Castor et al., 2013). SLX1 catalyzes the initial incision and MUS81 introduces the second cut on the opposing strand (Wyatt and West, 2014). GEN1 is a member of the RAD2/XPG family and can only access and cleave recombination intermediates when the nuclear membrane breaks down (Rass et al., 2010). GEN1 first forms a dimeric complex that contains the two active sites and then performs a dual symmetric incision at HJs, generating nicked duplex products that can be ligated.

The SDSA pathway also begins with the generation of a D-loop structure like the HJ pathway but also includes DNA synthesis in the 3′-direction, which extends the heteroduplex (Daley et al., 2014). The translocating D-loop then collapses, and the other resected DSB end will anneal to this extended DSB end. Both ends will go through replicative extension and ligation, which generates non-crossover products.

**Figure 2:**
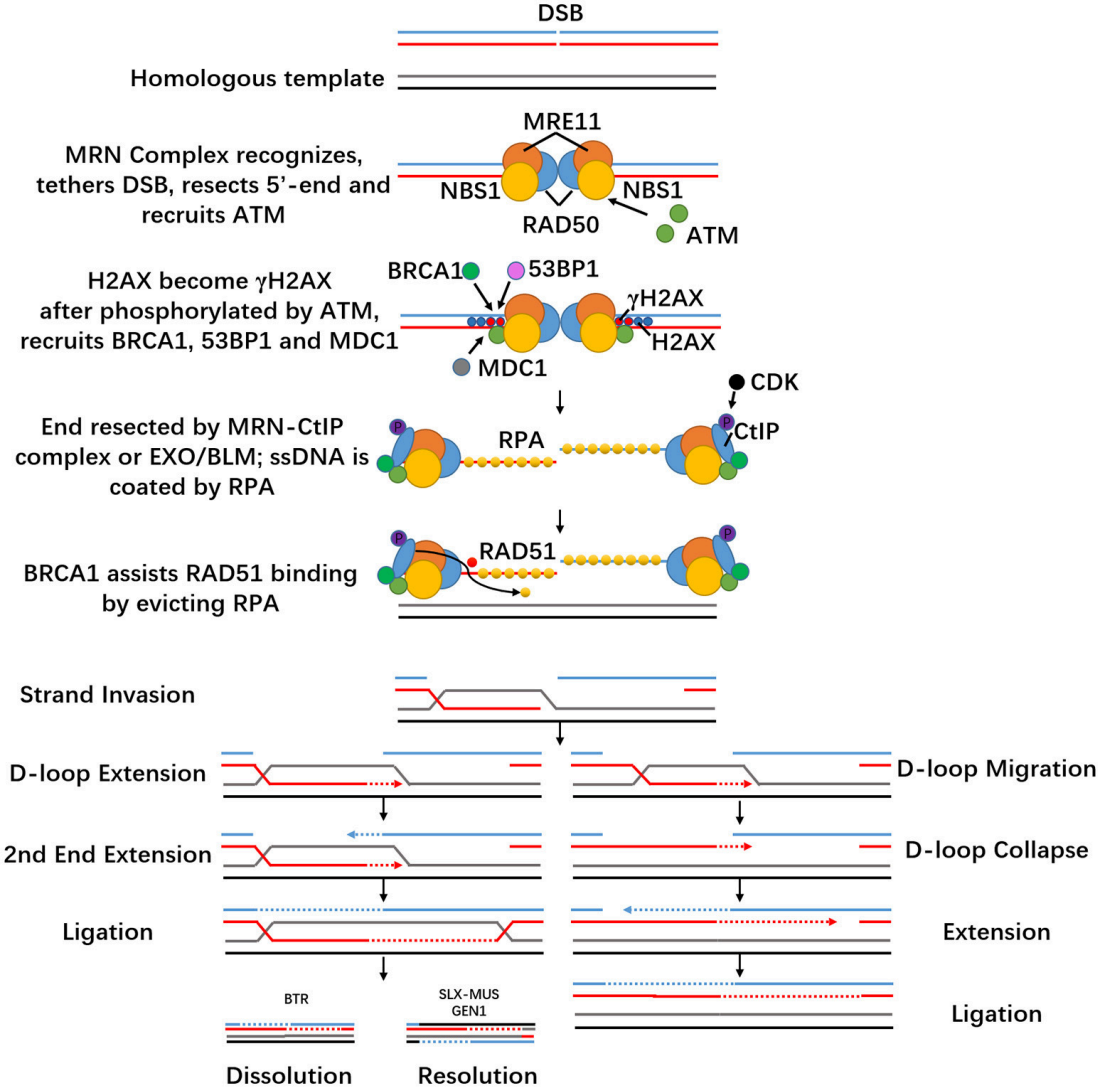
Homology-directed repair HDR. When DSB happens in the S- or G2-phase of the cell cycle and homologous sequence exists near the DSB, DSB can be handled through the HDR pathway if the ends of the DSB are resected. Ends will be coated with various proteins and then invade homologous duplex DNA to form an exchange intermediate: the D-loop structure. Most D-loop structures will be extended by DNA synthesis (dashed arrow). The second end pairs to the D-loop and starts extension. This pathway is called the double Holliday junction pathway. Ligation generates the characteristic double Holliday junction, which may be cleaved by HJ resolvases into either crossover or non-crossover products. The synthesis-dependent strand annealing pathway is illustrated on the right. After D-loop formation, replication and branch migration take place which can lead to D-loop translocation. The translocating D-loop is unstable and collapses easily. After collapse, the extended first end may anneal to complementary ssDNA in the resected second end. Replicative extension of both ends and ligation generates non-crossover products.

When it comes to single-stranded template repair (SSTR), the repair mechanism is quite different from the dsDNA repair template scenario. Richardson et al. found that human Cas9-induced SSTR requires the Fanconi anemia (FA) pathway, which was previously implicated in responses to interstrand crosslinks rather than nuclease-induced breaks (Richardson et al., 2018). They confirmed that SSTR is RAD51-independent while dsDNA donor HDR is RAD51-dependent. After FA pathway knockdown, the efficiency of SSTR decreased while simultaneously the levels of NHEJ increased, and the total editing stayed relatively constant. This means that the FA pathway can drive the repair events from NHEJ to SSTR. Additionally, FA pathway knockdown specifically inhibits SSTR and has no effect on NHEJ. RAD51C and XRCC3 are required for SSTR, but RAD51B and XRCC2 are not. They also found that FANCD2, a central player in the FA pathway, enriched even in the absence of an exogenous homology donor. In short, SSTR is much more efficient than HDR from a dsDNA donor but still needs future investigations. The HDR pathway is demonstrated in Figure 2.

## Favoring the HDR Pathway Using Chemical and Genetic Modulation

DSBs caused by Cas9 can go through both the NHEJ and HDR pathways, but in most cases they are handled by NHEJ (Frit et al., 2014), so it seems reasonable to inhibit key enzymes (e.g., DNA ligase IV) of the NHEJ pathway. Maruyama et al. (2015) investigated the effect of SCR7, a putative inhibitor of ligase IV, which targets the DNA binding domain of ligase IV, impeding its ability to bind to DSBs (Srivastava et al., 2012) in human epithelial (A549) and melanoma (MelJuSo) cell lines. Results showed that SCR7 promotes a 21 bp precise insertion (ssDNA donor with two 100 bp homology arms) in A549 cells 3-fold at 0.01 μM and 19-fold for MelJuSo at 1 μM. They also assessed the effect of SCR7 on insertion of a ~800 bp fragment (ssDNA donor with ~80 bp homology flanking sequence on both sides of the DSB) into a murine bone marrow-derived dendritic cell line (DC2.4 cells). After treating the DC2.4 cells with 1 μM SCR7, the efficiency of insertion increased by ~13-fold. It is worth mentioning that SCR7 affects lymphocyte development and can induce apoptosis. As additional approaches to DNA ligase IV inhibition, Chu et al. used the adenovirus 4 (Ad4) E1B55K and E4orf6 proteins to suppress NHEJ. These two proteins can mediate the ubiquitination and proteasomal degradation of DNA ligase IV (Forrester et al., 2011). Results showed that HDR efficiency was enhanced up to 7-fold (5 to 36%) by the Ad4 protein in human HEK293 cells. And in a mouse Burkitt lymphoma cell line, the addition of Ad4 proteins reduced transfection efficiency from 40 to 27%, but promoted HDR by 5-fold (Chu et al., 2015). Yu et al. identified two small molecules (L755507 and Brefeldin A) that could improve HDR efficiency. L755507, a β3-adrenergic receptor agonist, can increase HDR by 3-fold at 5 μM in mouse ESCs. Brefeldin A, an inhibitor of intracellular protein transport from the ER to the Golgi apparatus, promotes HDR by 2-fold at 0.1 μM in mouse ESCs (Yu et al., 2015).

Pinder et al. identified that RS-1 can enhance HDR between 3- and 6-fold, varying with the locus and transfection factor in HEK-293A human embryonic kidney and U2OS osteosarcoma cell lines (Pinder et al., 2015). RS-1 is a compound that stabilizes the association between Rad51 and DNA. They also found that an optimized ratio for Cas9/gRNA to homology donor plasmid doubled the HDR efficiency. Upon BRCA1 over-expression, HDR is increased by 2- to 3-fold.

Besides these inhibiting molecules, HDR can also be promoted using small interfering RNA (siRNA) to inhibit the expression of Ku protein, which is the pioneer protein in the NHEJ pathway. Li et al. assessed this method on pig fetal fibroblasts (Li et al., 2018). The result showed that by inhibiting Ku70 or Ku80 expression, HR can be promoted by 1.6- to 3-fold, as well as SSA and ssODN-mediated repairs. Yu et al. constructed a Rad51 and a Rad50 co-expression vector to evaluate its performance (Yu et al., 2011). It was determined that HR efficiency increased 110–245%. Chu et al. used short hairpin RNA (shRNA) in HEK293 cells to suppress key NHEJ pathway proteins such as KU70, KU80, and DNA ligase IV. Results showed that HDR efficiency was enhanced from 5 to 8–14% when transfected with single shRNAs against KU70, KU80, or DNA ligase IV. Furthermore, they found that the expression of the target gene diminished in the cells undergoing NHEJ blockade, which may be caused by local chromatin remodeling through extended DNA damage (Chu et al., 2015).

## Timed Delivery of the CRISPR-Cas9 System

While HDR is typically restricted to the S and G2 phases of the cell cycle, its efficiency can be increased by synchronizing and capturing cells at the S and G2 phases or using timed delivery. Lin et al. combined cell cycle synchronization techniques, using chemical inhibitors to arrest the cells at specific phases of the cell cycle with direct nucleofection of pre-assembled Cas9 ribonucleoprotein (RNP) in HEK293T cells (Lin et al., 2014). Results showed that using lower Cas9/RNP concentrations and cell cycle arrest can improve HDR efficiency to 31% (3.4-fold) at maximum. Consistent with this strategy, Yang et al. successfully enhanced HDR by three- to six-fold using the microtubule polymerization inhibitor nocodazole or ABT-751 (Yang et al., 2016). As for non-proliferating cells, BRCA1 is inhibited by the 53BP1 (Escribano-Diaz et al., 2013) and KEAP1-CUL3 complexes (Orthwein et al., 2015), and RIF1 thus will not bind to DSB. In addition, CtIP can function normally after being phosphorylated by CDK, but CDK is absent when cells stay in the G0/G1 phase (Escribano-Diaz et al., 2013). In order to overcome this inhibition, Orthwein et al. overexpressed mutated activated CtIP. This depleted the 53BP1 and KEAP1-CUL3 complexes simultaneously, which successfully activated the HDR pathway in G1 cells (Orthwein et al., 2015).

## Enhancing HDR by Using Overlapping Sequences

Several types of donor DNA have been used, such as plasmid DNA and synthetic oligonucleotides (Carroll and Beumer, 2014). Rational design of homology repair templates strongly enhances HDR efficiency (Renaud et al., 2016). By using a linear repair template with homologous flanks in zebrafish, HDR can increase by almost 10-fold (Irion et al., 2014). Chu et al. assessed the influence of the lengths of homology regions of the repair template on HDR efficiency (Chu et al., 2015). A homologous template is the most important component of HDR-mediated genome editing. It usually contains intended mutations or insertions flanked by homologous regions. Templates can be plasmids (up to kilobases modification) or single-stranded oligodeoxynucleotides (ssODN) (50–100 nt modification). It is suggested that sequencing around the interested region should be carried out because cell-specific mutations and single nucleotide polymorphisms (SNPs) can influence gRNA targeting up to 6-fold, merely one mismatch in 100 bases (Tham et al., 2016). This problem can be overcome by amplifying the homology arm from the genomic DNA extracted from target cells or by synthesize consulting sequence analysis (Salsman and Dellaire, 2017). It is important to remember that DSB should always be as close as possible to the region of homology, within 10 nt up and to a maximum of 100 nt (Elliott et al., 1998). Furthermore, having each homology arm about 50–100% the size of the payload that can promote HDR (Li et al., 2014).

## Enhancing HDR by Using Modified Cas9

As mentioned above, DSB ends must be resected so that they can enter the HDR pathway. CtIP, a key protein in early steps of DSB resection, is essential for HDR initiation. In order to ensure the presence of functional activated CtIP, Charpentier et al. fused a minimal N-terminal fragment of CtIP to the Cas9 protein (Charpentier et al., 2018). Forcing CtIP to the DNA cleavage site, through fusion to either catalytically dead Cas9 (dCas9) or Cas9 together with 800 bp homology arms, obtained a 2-fold increase in the efficiency of HDR in human fibroblast RG37DR cells, iPS cells, and rat zygotes. However, the expression of dCas9-CtIP is not sufficient by itself to stimulate integration. In addition, the patterns of indels induced by modified Cas9 were different from Cas9. This may be because the modified Cas9 induced a different balance of the end-joining pathway.

As for point mutation, current approaches to target base correction are inefficient and typically induce an abundance of random insertions and deletions at the target locus. Alexis et al. reported a powerful approach called a “base-editing (BE) system” to introduce specific point mutations without introducing DSB or a donor template by linking deaminases and dCas9 together (Komor et al., 2016). dCas9 helps deaminase to locate and deaminases modify cytidine to uridine. This conversion will then be repaired through various pathways (Hess et al., 2017). BE2 (optimized BE system) results from the addition of the uracil DNA glycosylase inhibitor, which increases the base-editing efficiency of the C>T substitution 3-fold (Hess et al., 2016). Additionally, the BE3 system was realized by changing the dCas9 to Cas9 D10A. This improvement achieved a 6-fold increase in efficiency over BE2 but exhibited a slightly increased indel frequency as nicks can lead to NHEJ at a low rate (Certo et al., 2011). Target-activation-induced cytidine deaminase (AID), a similar system, uses nickase Cas9 D10A to recruit the cytidine deaminase PmCDA1 to the target, achieving a mutation frequency of 35%. Adding UGI can obtain 2- to 3-fold increase in efficiency and reduction in deletions (Nishida et al., 2016). In addition, targeted AID-mediated mutagenesis (TAM) (Ma et al., 2016) and CRISPR-X (Hess et al., 2016) can generate transitions and transversions. These systems can both achieve the precision editing of single C>T bases with a low rate of indels as well as sequence diversification. By further Cas9 engineering, the inhibition of HR during the G1 phase will be overcome.

The CRISPR-Cas system has been fully studied and adapted for various applications over the decades, which gives us the ability to manipulate the genome as we desire. It has certain limitations, such as off-target effects (which can be overcame by rational sgRNA design; Doench et al., 2016) and low efficiency, which can be improved by utilizing the methodologies as described above. These promising strategies have proven their enhancement in the HDR pathway more than once with results varying from 2- to 30-fold. Combining various approaches can be a potential method of maximizing the rates of HDR. Here we only reviewed NHEJ inhibition by using inhibitors or hindering certain gene expression with siRNA or shRNA, CRISPR-Cas delivery in the G2/S phase, adding homologous arms in donor templets and using modified Cas9. These tactics surely make the CRISPR-Cas system more efficient. There is no doubt that more and more ways to boost CRISPR-Cas are imminent.

## Author Contributions

ML, WM, and DC designed the structure of this review. ML wrote the first version of the manuscript. SR, XT, KG, and QF helped to revise the manuscript. All authors have reviewed the final version of the manuscript.

### Conflict of Interest Statement

The authors declare that the research was conducted in the absence of any commercial or financial relationships that could be construed as a potential conflict of interest.
